# The effect of chelators on additives in the surface characterization and electrochemical properties of an eco-friendly electroless copper nano deposition

**DOI:** 10.1038/s41598-023-38115-8

**Published:** 2023-07-08

**Authors:** Suseela Jayalakshmi, Raja Venkatesan, Simon Deepa, Alexandre A. Vetcher, Sabah Ansar, Seong-Cheol Kim

**Affiliations:** 1 Department of Chemistry, School of Basic Sciences, Vels Institute of Science, Technology and Advanced Studies, Chennai, Tamil Nadu 600117 India; 2 School of Chemical Engineering, Yeungnam University, 280 Daehak-Ro, Gyeongsan, 38541 Republic of Korea; 3 Institute of Biochemical Technology and Nanotechnology, Peoples’ Friendship, University of Russia (RUDN), 6 Miklukho-Maklaya St., 117198 Moscow, Russia; 4 Complementary and Integrative Health Clinic of Dr. Shishonin, 5 Yasnogorskaya St, 117588 Moscow, Russia; 5 Department of Clinical Laboratory Sciences, College of Applied Medical Sciences, King Saud University, P.O. Box 10219, Riyadh, 11433 Saudi Arabia

**Keywords:** Chemistry, Materials science, Nanoscience and technology

## Abstract

We represent the results of a study on as the chelators used in the environmentally friendly electroless deposition bath changed depending on the amounts of hydroxides were present. The baths were prepared using polyhydroxides, glycerol and sorbitol, as chelators with copper methanesulfonate as the metal ion. Dimethylamine borane (DMAB) was used as the reducing agent with *N*-methylthiourea and cytosine, as additives in both the glycerol and sorbitol contained baths. Potassium hydroxide was used as the pH adjuster, with glycerol and sorbitol baths maintained at a pH of 11.50 and 10.75 respectively at a room temperature of 28 ± 2 °C. XRD, SEM, AFM, cyclic voltammetry studies, Tafel and Impedance studies, as well as additional methods, were employed to monitor and record the surface, structural, and electrochemical characteristics of the deposits and bath. The reports of the study gave interesting results, which clearly the effect of chelators on additives in the nano deposition of copper in an electroless deposition bath.

## Introduction

Surface coatings have found much importance in the modern world, where electroless plating has grabbed its own place in the industrial field over all other plating techniques^1–5^. In the present scenario copper, one of the oldest elements, because of its low electrical resistance and high electromagnetic migration has been widely used in electroless baths and has found applications in electrical, electronics, printing, food processing and many other industrial fields^6–8^. Electroless copper plating is used in the electronic industry, in the manufacture of flexible copper clad laminates (FCCL), the polyimide films coated with copper is used. In printed circuit boards, automobile parts etc., the copper deposited plastics such as polyethylene terephthalate (PET), Teflon, acrylonitrile butadiene styrene (ABS) are widely used. The copper coating is the best way to provide electromagnetic shielding, the electromagnetic interference shielding is an increasing demand to avoid interference between digital services. In microtechnology, copper and its alloys are used as interconnects and in packaging applications of ultra-large-scale integration (ULSI). With the fact that there are currently forty-four major deposition procedures available, the electroless coating method is one of the most straightforward methods because of its uniform coating on the edge and projection of any metal and non-metal surfaces^9^.

In an electroless bath, the chelating agent is the one which form stable complexes with the metal ion and hence enhances the rate of plating^10–12^. EDTA, the traditional chelator, because of its low biodegradability, must be replaced by an effective chelator with high biodegradability and stability^13,14^. In that concern, in recent days, the polyhydroxides attracted the electroless baths by its effective chelating properties in alkaline medium^15^. Polyhydroxylic alcohols are bio-degradable and, in alkaline medium, they possess very good chelating property. The electroless copper plating solutions containing these chelators are stable and, under the optimal conditions selected, copper coatings up to 3 µm thickness can be obtained in 1 h at ambient temperatures. Many naturally occurring polyhydroxylic alcohols such as xylitol, D-mannitol, erythritol, alditol, adonitol, glycerol, D-sorbitol, maltitol etc. are used as the complexing agent in the recent studies. In this work, polyhydroxylic compounds such as glycerol and sorbitol are used as the complexing agents. The traditional reductant formaldehyde, since proved to be carcinogenic, a non-formaldehyde reductant DMAB has gained importance in the study^16,17^. They possess high solubility, varying pH tolerance and workability over varying temperatures. The role of additives, temperature, and pH in electroless baths are unforgettable^18–21^.

The copper electroless bath contains methanesulphonic acid as the bath solution as it produces high quality deposition layers of even distribution^22–24^. The copper methanesulfonate electroless bath in this study contains polyhydroxides, glycerol and sorbitol as chelators and DMAB as the reducing agent. Potassium hydroxide is used as the pH maintainer at a room temperature of 28 ± 2 °C. *N*-methylthiourea and cytosine are used as the bath additives at a concentration of 1 ppm in both glycerol and sorbitol contained baths. Two additives *N*-methylthiourea, and cytosine used, completely changed and introduced new interesting leads to the physical and electrochemical results of the bath study.

## Materials and methods

### Chemicals

The chemicals were procured from the sources mentioned and used as such without further purification. Ethanol, Ammonia solution (Fisher), Copper methanesulfonate (S.D. Fine Chemicals), Copper carbonate (Merck), Glycerol, and Sorbitol (Fisher), *N*-methylthiourea, and Cytosine (S.D Fine Chemicals), Potassium hydroxide (Sigma-Aldrich), and Dimethylamine borane (DMAB) (Merck). All stock solutions were prepared using double distilled water.

### Preparation of eco-friendly electroless deposition bath

The electroless deposition baths were prepared with copper methanesulfonate as the metal ion, glycerol, and sorbitol as chelators, DMAB as the reducing agent, and KOH as pH adjuster with the added stabilizers. The electroless plating was carried out on an epoxy substrate^25,26^. The substrate surface was polished with grit paper and rinsed with distilled water. The surface etching is done using a solution of KMnO_4_ and H_2_SO_4_, to remove any oxidized surface layer. It is then sensitized with SnCl_2_ solution (SnCl_2_ mixed with HCl) and activated with HCl solution of PdCl_2_ to improve the deposition rate and the adhesive properties of the Cu thin film. In a 100 ml beaker, pre-treated and pre activated epoxy sheet (2.0 cm × 2.0 cm × 0.1 cm) was dipped in the bath solution for the plating process for a period of 1 h. The composition of the electroless deposition baths is shown in Table 1. GPB, GMtu, GCyt, SPB, SMtu, and SCyt, respectively, are abbreviations for glycerol plain bath, glycerol + *N*-methylthiourea, glycerol + cytosine, sorbitol plain bath, sorbitol + *N*-methylthiourea, and sorbitol + cytosine.

**Table 1:** Bath composition and condition of copper methanesulfonate baths with stabilizers.

Bath contains	Glycerol (g/L)			Sorbitol (g/L)		
	Plain bath (GPB)	*N*-methylthiourea (GMtu)	Cytosine (GCyt)	Plain bath (SPB)	*N*-methylthiourea (SMtu)	Cytosine (SCyt)
CuMS (II) ion contacting salt	3 g/L	3 g/L	3 g/L	3 g/L	3 g/L	3 g/L
Glycerol/Sorbitol	20 ml/L	20 ml/L	20 ml/L	20 ml/L	20 ml/L	20 ml/L
Reducing agent	5 g/L	5 g/L	5 g/L	5 g/L	5 g/L	5 g/L
KOH (pH)	11.50	11.50	11.50	10.75	10.75	10.75
Temperature (± 2 °C)	28 °C	28 °C	28 °C	28 °C	28 °C	28 °C
Stabilizers	0 ppm	1 ppm	1 ppm	0 ppm	1 ppm	1 ppm

### Calculation of deposition rate and thickness of copper deposits

The rate of deposition of the electroless copper deposits can be calculated from the relation.

1 $${\text{Rate}}\;{\text{of}}\;{\text{deposition}}\;(\upmu {\text{m/h)}} = {\text{Thickness}}/{\text{Deposition}}\;{\text{time}}$$

The thickness of the copper deposits can be calculated from the following relation,

2 $$\mathrm{T}=\mathrm{W}\times 1{0}^{4}/\mathrm{dAt}$$

where ‘W’ is the mass of the deposit (g), ‘d’ is the density of the film material (8.96 g/cm^3^), ‘A’ is the area of the film coated (cm^3^) and ‘t’ is the coating duration (h).

### Characterization

#### X-ray diffraction

In the electroless deposition process, the structural properties of the deposited copper are tested by XRD analysis at room temperature using an analytical X-ray diffractometer (XRD; Mini Flex 120 II-C). For the preparation of the samples were placed on a glass slide, and the spectrum was recorded using CuKα as the radiation source (λ = 0.15406 nm). Each scan is performed at a rate of 10 steps per degree, with a diffraction angle ranging from 20.0° to 80.0°.

The crystallite size of the copper deposits is calculated using Debye Scherrer’s equation^27,28^.

3 $${\text{D}} = {\text{K }}\uplambda /\upbeta \;\cos \uptheta$$

where K—Scherrer constant; λ—Wavelength of light used for the diffraction; β—Full width at half maximum of the sharp peaks; θ—angle measured.

Specific surface area of the copper deposits is calculated by the formula;

4 $${\text{S}} = 6 \times 10^{3} /{\text{dDS}}$$

where D is the crystallite size (nm); d—theoretical density of copper (8.96 g/cm^3^).

#### Scanning electron microscopy

SEM was used to study the morphological characterization of the deposited copper (*S-4800, Hitachi, Japan*). Using a dried samples were placed on a metal stub with double-sided carbon tape and sputtered with 10 nm gold–palladium. Images were recorded at a 20 kV accelerating voltage.

#### Atomic force microscope

The surface roughness of the copper deposits was revealed by an AFM with 200 kV accelerating voltage. The AFM used for this study was Park XE-100, Germany. A resolution of 10 pm can be achieved and samples in air and in liquids can also be analyzed by this method.

#### Cyclic voltammetry

The electrochemical properties of copper deposits samples are tested by the cyclic voltametric analysis. The quantity and quality of the deposits were described by the anodic peak potential and peak current values^29,30^. Electrochemical studies were carried out using an electrochemical workstation (Autolab PGSTAT) with an aqueous 1 M H_2_SO_4_ electrolytes. The three-electrode arrangement was made using platinum wire as the counter electrode, Ag/AgCl in KCl as the reference electrode and the standard glassy carbon electrode is used as the working electrode. The adsorption, diffusion and the mechanism of the homogenous coupled chemical reaction can be understood from the width, amplitude, and the potential of the voltammogram peaks.

#### Tafel polarization

In the Tafel polarization method, the potential of the working electrode is changed, and the current produced as a function of time or potential is monitored. The metal penetration rate or the rate of loss of weight per unit area is the measure of the rate of corrosion. The corrosion current obtained from the Tafel plot can be related to the rate of corrosion^30,31^. The polarization resistance obtained from the slope of linear portion of the plot is related to the corrosion current density as follows.

5 $${\text{R}}\rho = \frac{{\upbeta _{{\text{a}}} \times \upbeta _{{\text{c}}} }}{{2.303\left( {\upbeta _{{\text{a}}} + \upbeta _{{\text{c}}} } \right){\text{I}}_{{{\text{Corr}}}} }}$$

where Rρ is the polarization resistance, βa and βc are Tafel slopes magnitude of anodic and cathodic Tafel lines. The rate of corrosion is directly proportional to the corrosion current obtained from the Tafel plot.

The deposition rate can be calculated from the deposition current values obtained from the Tafel plots. The standard ASTM (American society for testing and materials) equation can be used to calculate the deposition rate.

6 $${\text{Deposition}}\;{\text{rate}}\;\left( {\upmu {\text{m}}/{\text{h}}} \right) = 3.73 \, \times 10^{ - 4} \left[ {{\text{i}}_{{{\text{dep}}}} /{\text{D}}} \right] \times {\text{Eq}}{\text{.wt}}$$

where, i_dep_ = Deposition current; D = Density of the copper metal (g/cm^3^); Eq. wt. = Equivalent weight of the copper metal (g).

#### Electrochemical impedance spectroscopy

The corrosion phenomenon of an electroless bath can be studied using the AC impedance technique^32^. The characterization of metal coated surfaces can be done with this powerful technique^33,34^. The real and the imaginary components of the impedance response is recorded. The shape of the EIS spectrum, circuit parameters and the circuit description code are used to find the inductance, charge transfer resistance and double layer capacitance values. The voltage drop at the interface of the working electrode and the electrolyte is noted by applying a voltage between the working electrode and the counter electrode. Thus, the interfacial charge transfer between the electrolyte and a conductor (working electrode) is obtained.

7 $${\text{L}}_{1} + {\text{R}}_{1} + \frac{{{\text{C}}_{2} }}{{{\text{R}}_{2} }} + \frac{{{\text{Q}}_{3} }}{{{\text{R}}_{3} }}$$

where L_1_—Inductance; C_2_—Double layer capacitance; R_1_, R_2_, and R_3_—Resistances; Q_3_—Constant phase element (imperfect capacitor).

### Ethical approval

All author declared that all the ethical standard required for the preparation and publication are complied.

### Consent to participate

All person named as author in this manuscript have participated in the planning, design and performance of the research and in the interpretation of the result.

## Results and discussion

### Effect of conditions of bath parameters on electroless deposition bath

**Figure 1:**
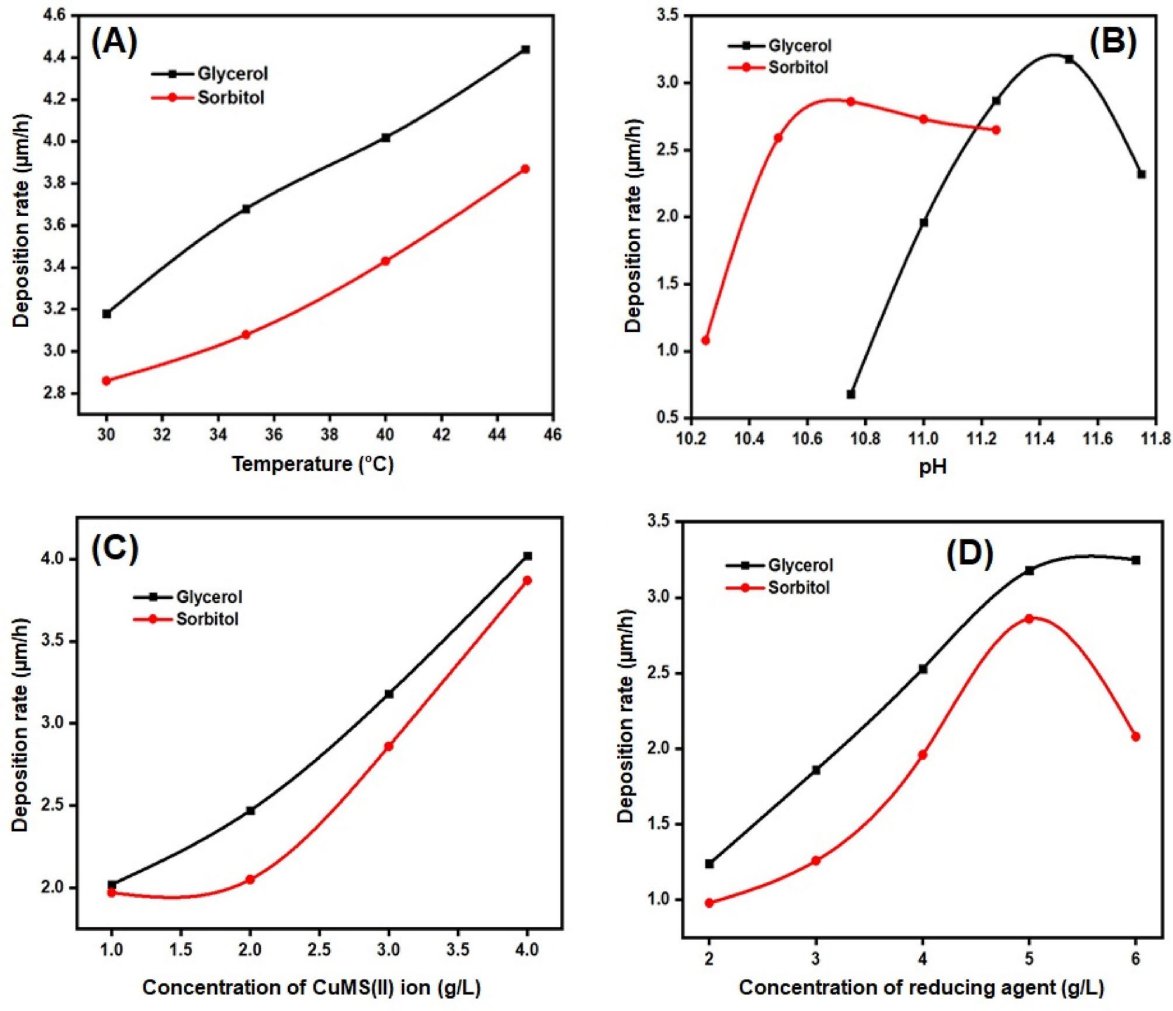
Rate of deposition of copper in glycerol and sorbitol contained baths; (**A**) Effect of temperatures; (**B**) Effect of pH; (**C**) Concentration of CuMS (II) ion; and (**D**) Concentration of reducing agent.

The optimization of an electroless deposition bath, which is accomplished and estimated using a straightforward weight gain approach, greatly depends on bath factors such as metal ion concentration, reducing agent, pH, and temperature. Figure 1A shows an increase in deposition rate with the increase in temperature of the electroless deposition bath. Similar effects have been observed in Fig. 1C, with the increase in concentration of the Copper ion solution the deposition rate increases. However, a non-linear characterization was seen in Fig. 1B,D, with increase in the pH of the bath and concentration of DMAB, the deposition rate first increases and then shows a gradual decrease which further led to the decomposition of the deposition bath. In general, higher deposition rates have been achieved by electroless baths maintained at higher temperatures i.e., above 45 ℃. But in this study, the electroless deposition bath is maintained at room temperature where the bath is found to be more stable.

The stability and rate of deposition of the electroless baths are significantly influenced by the pH of the bath. By adjusting the pH of the baths that included glycerol and sorbitol, they were optimized through a process of trial and error. Finally, the pH of the sorbitol bath was tuned at 10.75, whereas the pH of the glycerol bath was 11.50. The concentration of copper ion as a function of deposition rate clearly indicates that the deposition increases with the increase in concentration. Here the stability of the bath highlights, only at a concentration of 3 g/L of copper ion, the deposits were uniform, smooth which has been reflected in the bath stability.

### Surface morphology and property of the deposits

#### XRD analysis

**Figure 2:**
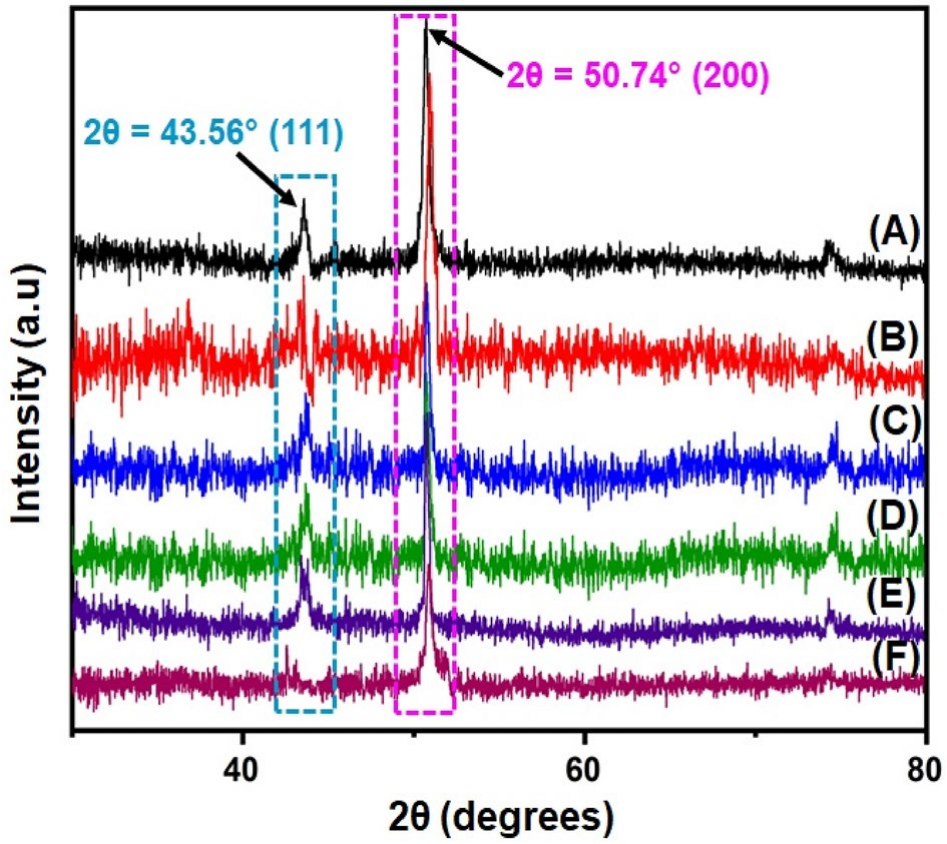
XRD pattern of copper deposits of electroless deposition baths; (**A**) GPB, (**B**) GMtu, (**C**) GCyt, (**D**) SPB, (**E**) SMtu, and (**F**) SCyt.

The structural characteristics of the copper deposits were analyzed using the XRD studies. The crystallite size of the deposits calculated from the Debye Scherrer’s equation is proportional to the inhibiting nature of the copper deposits. The copper deposits are arranged by the copper methanesulfonate in the electroless bath because to its high solubility and high conductivity. The X-ray diffraction peaks of (200) and (111) planes of copper deposits in the electroless deposition baths are shown in Fig. 2.

The crystallite size of deposits obtained from glycerol plain bath (GPB) is found to be 20.17 nm with a specific surface area of 33.19 m^2^/g. The deposits of GMtu and GCyt contained bath has shown a crystallite size of 19.90 and 19.26 nm respectively with a specific surface area of 33.65 and 34.76 m^2^/g. The crystallite size of sorbitol plain bath (SPB) is found to be 25.55 nm with a specific surface area of 26.20 m^2^/g. While that of SMtu and SCyt contained electroless deposition baths has shown crystallite size of 26.99 and 26.08 nm with a specific surface area of 34.76 and 26.20 m^2^/g, which indicates that the deposits of glycerol contained baths are best with the *N*-methylthiourea and cytosine added as additives acted as accelerators. While the same additives added in the sorbitol contained baths shown results which indicates, they acted as inhibitors exhibiting performance less than the sorbitol plain bath.

#### SEM analysis

**Figure 3:**
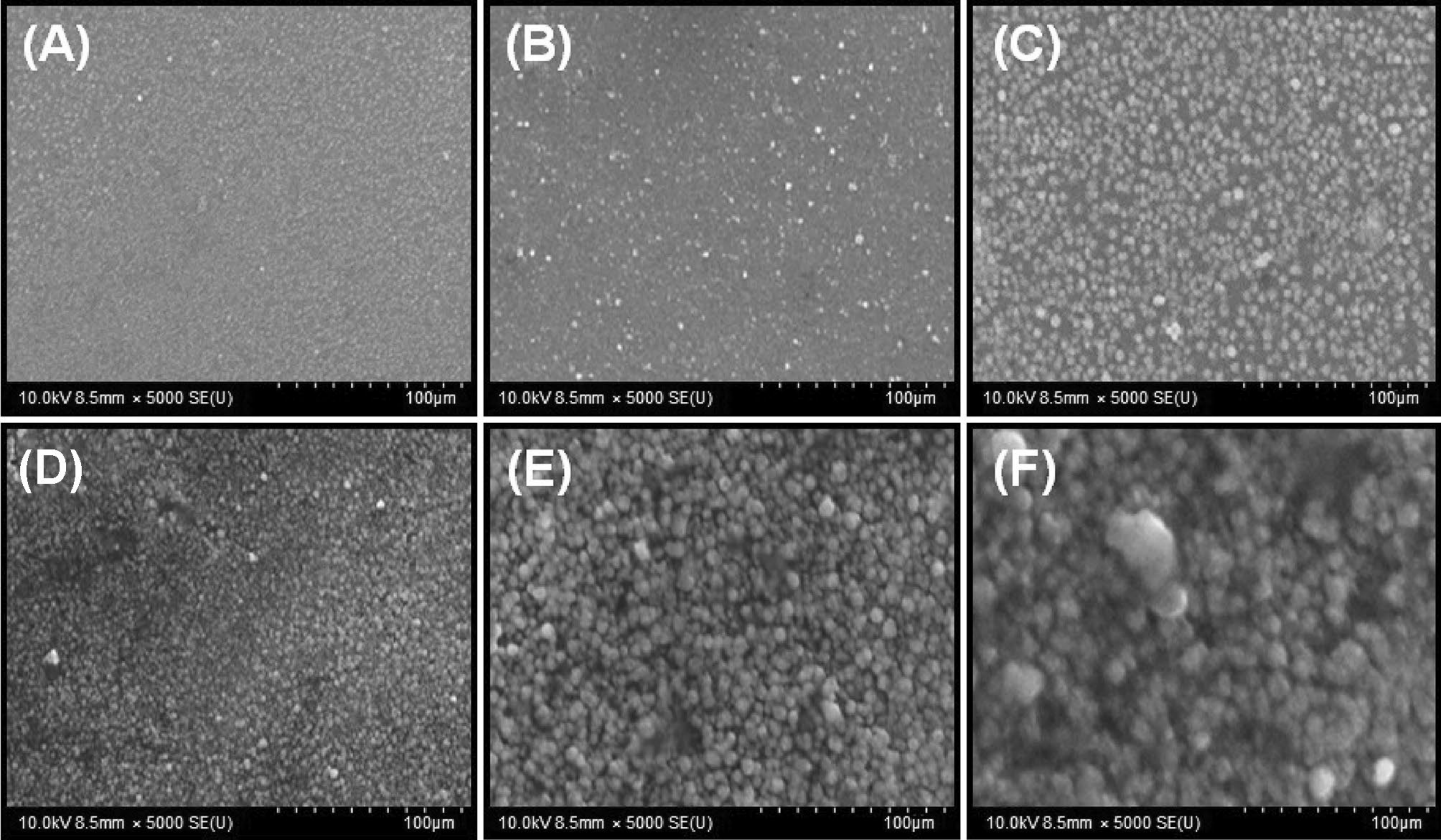
SEM images of copper deposits of electroless deposition baths; (**A**) GPB, (**B**) GMtu, (**C**) GCyt, (**D**) SPB, (**E**) SMtu, and (**F**) SCyt.

With the help of SEM, the surface morphology of copper deposits in the electroless deposits was studied; the results of this study are shown in Fig. 3. As seen in Fig. 3A, the surface morphology of copper deposits showed a spherical rounded shape. Figure 3B,C show the rough rounded structure of the GMtu and GCyt deposits. The activation of novel cluster sites has been greatly decreased or prevented by copper deposition. Figure 3D provides clear evidence of the microstructural perfection and nodularity removal. This could be the reason of the very smooth and bright quaternary electroless deposit. This was further confirmed by studying the morphology of the SMtu and SCyt deposits, which are shown in Fig. 3E,F.

According to Fig. 3A, the copper substrate has an even distribution of a few small and compact GPB is for deposition. There are a few pores, though, demonstrating that the GPB coating completely encircle the copper substrate surface. The copper substrate is entirely covered by the electroless GPB coating as the deposition time increases, and the crystal grain gradually grows up. For instance, the GMtu and GCyt coatings used on copper plates in Fig. 3B,C were compact and smooth, with a spherical structure that has been confirmed by other literature. In contrasted with the smooth deposition surface of the SPB deposit (Fig. 3D), the nodular structure of the SPB, SMtu, and SCyt deposits is clearly evident in Fig. 3D–F.

#### AFM analysis

**Figure 4:**
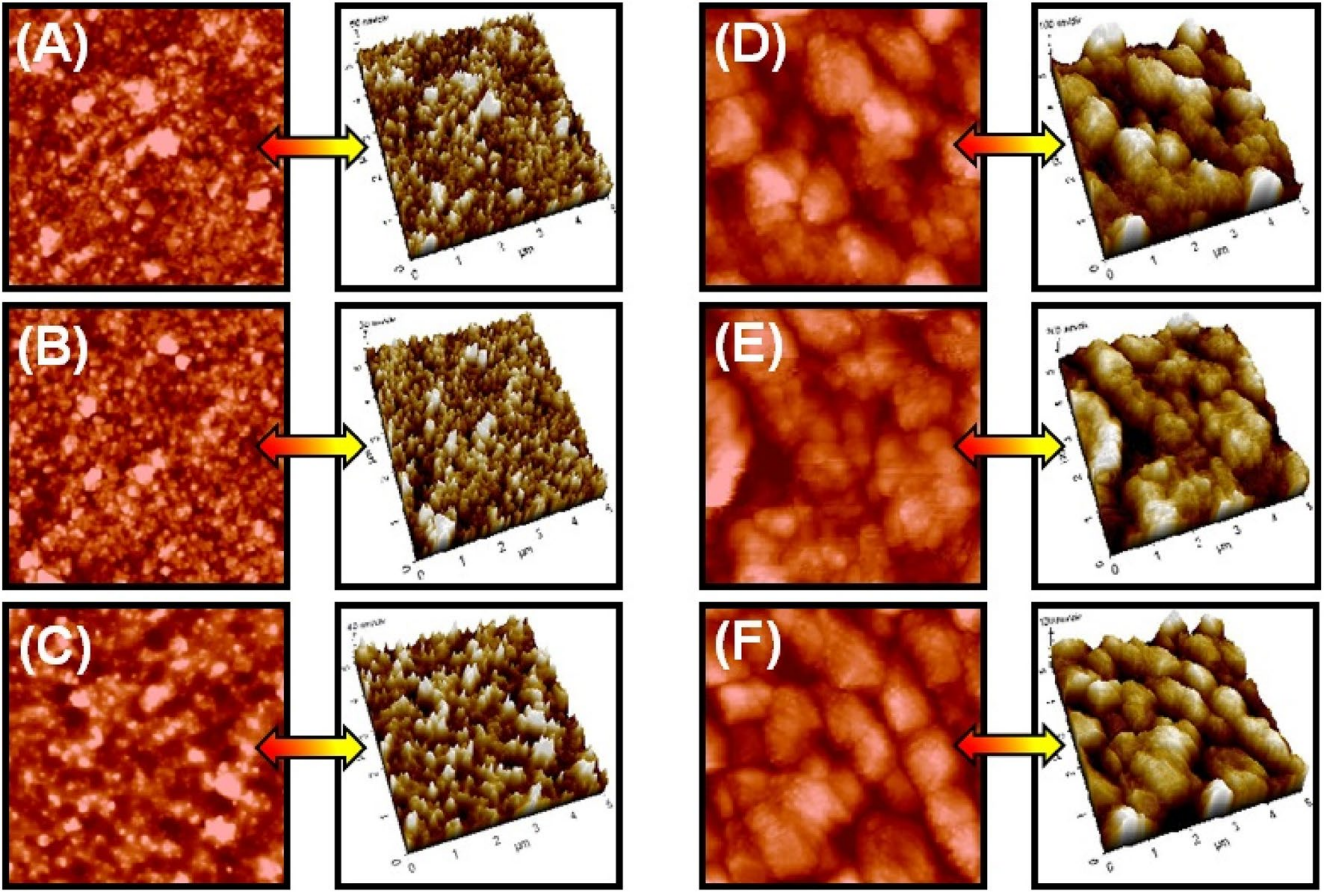
2D and 3D AFM images of the copper deposits of electroless deposition baths; (**A**) GPB, (**B**) GMtu, (**C**) GCyt, (**D**) SPB, (**E**) SMtu, and (**F**) SCyt.

The roughness value obtained from the AFM studies is inversely proportional to the smoothness of the deposits. Figure 4, show the roughness values and the shapes of deposits of the electroless deposition baths. The deposits of copper obtained from the glycerol plain bath (GPB) are of small grain sized with a roughness value of 23.41 nm. The deposits of GMtu and GCyt contained bath has given coarse sand and gravel sized deposits with a roughness value of 24.52 and 23.28 nm respectively. While the copper deposits of sorbitol plain bath (SPB) and SMtu contained electroless baths exhibited coarse sand deposits and the SCyt bath has shown honey comb deposits with a roughness value of 60.09, 65.31 and 53.55 nm respectively. The results clearly indicates that the deposition bath containing glycerol as the chelator has shown smooth deposits when compared to the deposition bath containing sorbitol.

### Electrochemical characteristics of the deposition baths

**Figure 5:**
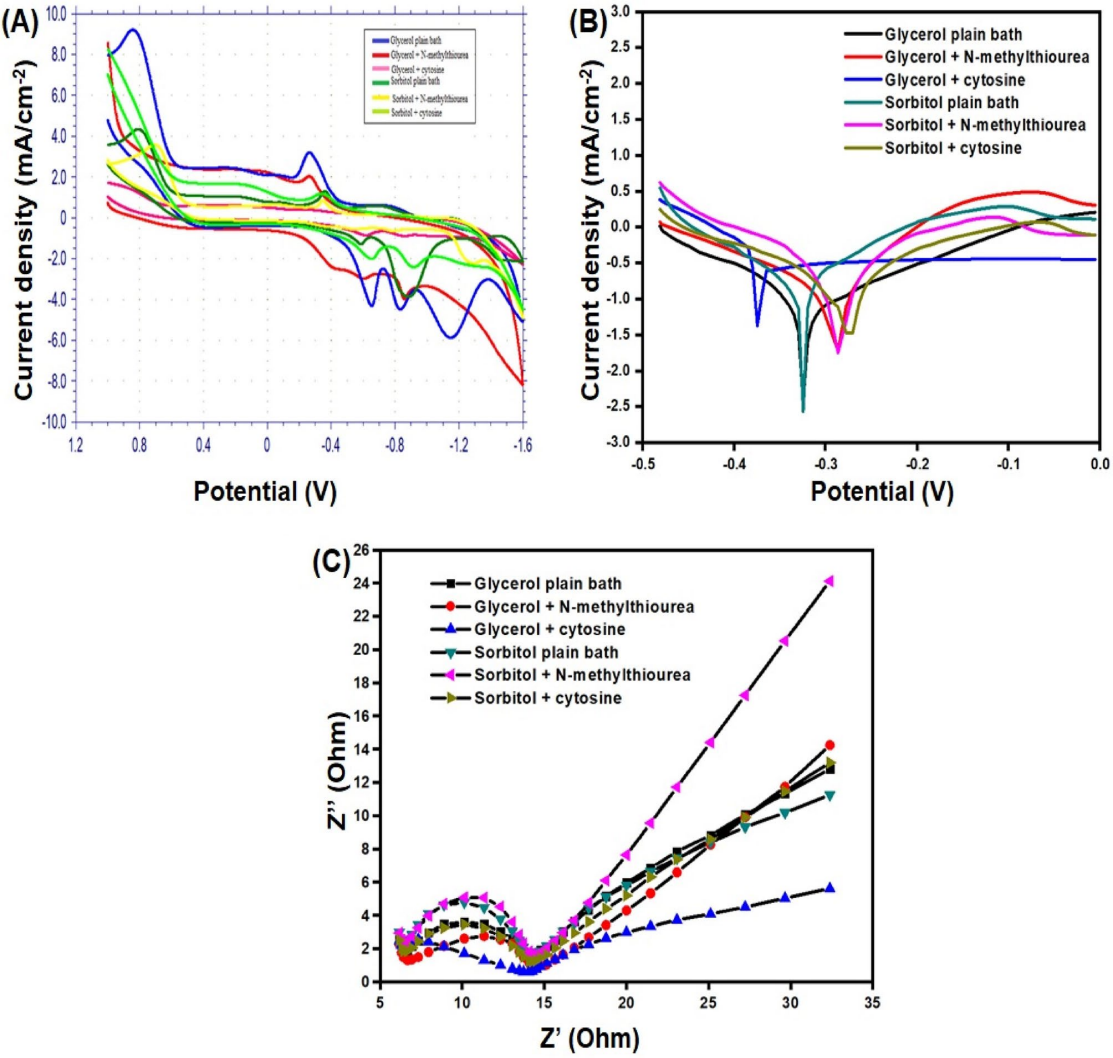
(**A**) Cyclic voltammogram of electroless deposition baths with stabilizers; (**B**) Tafel polarization curve of electroless deposition baths; and (C) Nyquist diagram of electroless deposition baths.

The cyclic voltametric tests demonstrated in Fig. 5A can be used to evaluate the electrochemical characteristics as well as the amount and quality of the electroless deposits. The anodic peak potential and the anodic peak current talks about the accelerating and inhibiting nature of the additives added in the bath. The results clearly indicate that the additives acted both as accelerator and inhibitors, depending on the nature of the polyhydroxides in the electroless baths. The tafel polarization studies gives the deposition rate as well as the corrosion current.

The rate of corrosion can be directly related to the corrosion current. Figure 5B it is clearly apparent that, compared to a bath containing sorbitol, a bath containing glycerol shows excellent deposition and corrosion resistance characteristics. Table. 2 show the corrosion current and the deposition rate of all the six electroless deposition baths. The deposition rate of glycerol was found to be 3.1 µm/h, and that of *N*-methylthiourea and cytosine contained bath was found to be 3.3 µm/h and 4.1 µm/h respectively which shows that the additive contained glycerol baths has shown performance better than its own parent bath. The deposition rate of sorbitol plain bath, *N*-methylthiourea contained bath and cytosine contained bath was found to be 2.3 µm/h, 1.6 µm/h and 1.1 µm/h respectively which shows that the performance of *N*-methylthiourea and cytosine contained bath is less than the sorbitol plain bath which is apparently clear that the additives acted as inhibitors in the sorbitol bath. The charge transfer resistance obtained from the electrochemical impedance studies clearly falls in accordance with all the other studies which clearly shows that, the bath containing glycerol given the best results while compared to that of the sorbitol complexed baths. Figure 5C shows the EIS curves of copper deposits of electroless deposition baths. Table 3 lists the results obtained from the EIS studies.

**Table 2:** Tafel polarization values and deposition rate for electroless copper methanesulfonate sorbitol plain bath with stabilizers.

S. no	Electroless bath with stabilizers (1 ppm)	β_a_ mV/decade	β_c_ mV/decade	E_corr_ (mV)	I_corr_ (mA)	Deposition rate (µm/h)
1	GPB	309.1	156.3	− 460.8	235.3	3.1
2	GMtu	379.2	113.3	− 536.9	251.7	3.3
3	GCyt	155.2	237.2	− 454.0	316.6	4.1
4	SPB	352.3	93.4	− 500.9	177.8	2.3
5	SMtu	365.5	357.7	− 401.6	127.1	1.6
6	SCyt	198.5	170.9	− 360.0	86.9	1.1

**Table 3:** Electrochemical impedance value for electroless copper methanesulfonate bath with stabilizers.

S. no	Glycerol plain bath with stabilizers (1 ppm)	Inductance (H/cm^2^)	Double layer Capacitance (C_dl_) (µF/cm^2^)		Charge transfer Resistance (Rt) (mΩ/cm^2^)		
		L_1_ × 10^–6^	C_2_ × 10^–6^	C_3_ × 10^–3^	R_1_	R_2_	R_3_
1	GPB	− 0.382	0.496	1.041	11.24	11.04	67.86
2	GMtu	− 0.323	0.828	0.812	4.00	4.33	59.66
3	GCyt	− 0.334	0.938	1.081	5.46	4.74	59.13
4	SPB	− 0.359	0.545	6.118	8.19	6.89	94.82
5	SMtu	− 0.382	0.493	0.487	11.24	10.93	107.80
6	SCyt	− 0.347	0.895	1.458	7.46	5.37	102.94

## Conclusion

The surface characteristics of the deposits from SEM analysis revealed that the copper deposits obtained from the glycerol contained baths are much dense, compact, and finer than the deposits obtained from the sorbitol contained baths. The roughness values obtained from the AFM studies also stands in agreement with the results obtained from SEM studies. The structural characteristics of the deposits from XRD clearly indicates that the crystallite size of the deposits obtained from the glycerol contained baths are lesser than the deposits obtained from the sorbitol contained baths. Therefore, the chelating property of glycerol is found to be better than sorbitol, in the electroless copper baths. The electrochemical characteristics of the deposition bath divulges the behavioral changes of additives as accelerators and inhibitors and their transition depending on the nature of the chelators in the bath. The surface, structural and electrochemical results also articulate, the influence of number of hydroxides presents in the chelator, on the deposition process. The studies suggest that the additives *N*-methylthiourea and cytosine, which acted as accelerator in the glycerol contained bath, behaved differently as inhibitors in the sorbitol used baths. Therefore, it is evident from the study that the trihydroxylic chelator, glycerol had much influence on the deposition bath compared to the hexahydroxylic chelator, sorbitol.

## Data Availability

All data generated or analyzed during this study are included in this published article.
